# pH-Controlled Synthesis of Nb_2_O_5_ Nanoparticles with Enhanced Performance for Photodegradation of Organic Pollutants and H_2_ Production

**DOI:** 10.1021/acsphyschemau.5c00132

**Published:** 2026-03-18

**Authors:** Carolina X. J. Silva, Marcela Piassi Bernardo, Ivan José Silva, Sheila Cristina Canobre, Osmando F. Lopes

**Affiliations:** Institute of Chemistry, Federal University of Uberlândia (IQUFU), Avenue João Naves de Ávila, 2121, Santa Monica, 38400-902 Uberlândia, Minas Gerais, Brazil

**Keywords:** niobium pentoxide, hydrothermal synthesis, photocatalysis, green hydrogen production, surface hydroxyl groups

## Abstract

Niobium pentoxide (Nb_2_O_5_) is a promising photocatalyst for environmental remediation and solar hydrogen production; however, the role of synthesis parameters in governing its phase composition and charge-carrier dynamics remains insufficiently understood. Herein, we systematically investigate the effect of synthesis pH (2, 4, and 6) on the structural, morphological, electronic, and photocatalytic properties of Nb_2_O_5_ nanoparticles prepared via two hydrothermal routes. pH variation in Route 1 promotes a phase transition from pseudohexagonal TT-Nb_2_O_5_ (pH 2) to hydrated Nb_2_O_5_ *n*H_2_O (pH 4–6), accompanied by pronounced morphological evolution from nanoneedles to lamellar structures. In contrast, Route 2 preserves the TT-Nb_2_O_5_ phase regardless of post-treatment pH, highlighting its structural stability. In organic pollutant degradation, all samples were active toward Rhodamine B and amiloride, where mechanistic studies revealed a hole-dominated oxidation pathway with secondary involvement of superoxide radicals. Hydrated Nb_2_O_5_·*n*H_2_O phases exhibited strong adsorption capacity, whereas TT-Nb_2_O_5_ showed higher intrinsic photocatalytic efficiency. Stability tests confirmed sustained activity over four cycles. TT-Nb_2_O_5_ materials also exhibited superior charge-transfer properties and were active in H_2_ evolution, achieving up to 4104.5 μmol g^–1^ after 8 h (Nb_2_O_5_-AP2). Optimization of Pt loading revealed an optimal cocatalyst content of 0.5 wt %, while long-term assays confirmed stable H_2_ production over 24 h of operation. Photoluminescence results indicated that recombination suppression contributes to performance enhancement but does not solely govern activity. These findings demonstrate that synthesis pH governs phase formation, surface chemistry, and charge-carrier dynamics in Nb_2_O_5_, providing fundamental insights into the rational design of niobium-based photocatalysts tailored for integrated environmental and energy applications.

## Graphical abstract

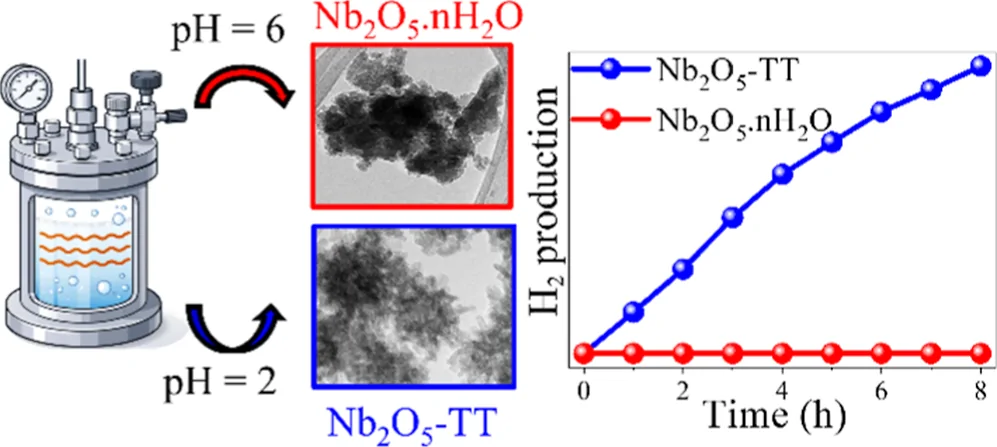

## 1 Introduction

The growing demand for clean energy and effective environmental remediation technologies has intensified the search for sustainable strategies capable of simultaneously addressing both challenges. In recent years, several catalytic approaches have been explored for pollutant degradation and hydrogen production, including electrocatalysis,^1^ piezocatalysis,^2^ and photocatalysis.^3^ Electrocatalytic systems offer high controllability and efficiency but often require external electrical input, increasing operational costs and complex infrastructure.^4,5^ Piezocatalysis, which converts mechanical energy from vibrations or friction into chemical energy, has emerged as an alternative route for pollutant degradation and H_2_ generation.^2,6–8^ Despite its conceptual appeal and recent progress, its practical application remains limited by relatively low energy-conversion efficiencies and dependence on continuous mechanical stimulation.^9^ In this context, photocatalysis stands out as a particularly attractive strategy, as it directly harnesses solar or artificial light to drive redox reactions under mild conditions.^10^

Photocatalytic processes enable the degradation of persistent organic pollutants while simultaneously producing green hydrogen, offering a dual environmental benefit and contributing to carbon-neutral energy cycles.^11–13^ Therefore, the development of efficient, stable, and cost-effective photocatalytic materials remains a central objective for advancing integrated environmental and energy solutions. In particular, niobium pentoxide (Nb_2_O_5_) has emerged as a promising photocatalyst due to its nontoxicity, chemical and thermal stability, abundance in nature (notably in Brazil), and favorable band gap for UV and near-visible light activation.^14–16^ Moreover, Nb_2_O_5_ can be synthesized in different polymorphs, such as pseudohexagonal TT-Nb_2_O_5_ and hydrated Nb_2_O_5_·*n*H_2_O, which show distinct structural and photocatalytic properties.^17,18^ These features make Nb_2_O_5_ a versatile platform for environmental and energy applications.

Despite these promising features, a key challenge in advancing Nb_2_O_5_ photocatalysts lies in the lack of systematic studies evaluating how synthesis parameters affect its performance. Filho et al.^19^ observed that increasing the calcination temperature led to the removal of surface hydroxyl groups, which are crucial for photocatalytic activity. Samples calcined at higher temperatures exhibited a significant decrease in photocatalytic efficiency, attributed to the loss of these hydroxyl groups, which facilitate the generation of reactive oxygen species essential for pollutant degradation. This finding underscores the pivotal role of surface hydroxyl groups in enhancing the photocatalytic processes of Nb_2_O_5_ material. Among these parameters, pH is particularly critical, as it influences hydrolysis, nucleation, and crystal growth during synthesis.^20,21^ While several studies have focused on the evaluation of temperature synthesis,^22^ doping strategies,^23,24^ and composite formation,^25–27^ the direct effect of synthesis pH on the phase composition, morphology, and photocatalytic activity of Nb_2_O_5_ remains underexplored. A deeper understanding of how pH mediates the formation of TT-Nb_2_O_5_ or Nb_2_O_5_ *n*H_2_O phases, and how these structural differences impact pollutant degradation and hydrogen evolution, is essential for the rational design of efficient Nb_2_O_5_-based materials.

In this work, the effect of the synthesis pH was systematically investigated on the properties of Nb_2_O_5_ nanoparticles prepared via two hydrothermal routes. Using a suite of characterization techniques such as, XRD, Raman, frontier Fourier transform infrared (FTIR), X-ray photoelectron spectroscopy (XPS), UV–vis DRS, scanning electron microscopy (SEM), transmission electron microscopy (TEM), and BET, we examined structural, electronic, and morphological differences as a function of pH synthesis. The photocatalytic performance of the synthesized materials was assessed through the degradation of dye and drug, Rhodamine B (RhB) and Amiloride, as well as hydrogen production under UV–vis light in the presence of methanol as a sacrificial agent. Our findings demonstrate the critical role of pH in directing phase formation and surface characteristics and underscore the importance of synthetic control for optimizing Nb_2_O_5_ photocatalysts for environmental remediation and green energy generation.

## 2 Materials and Methods

### 2.1 Synthesis of Nb_2_O_5_ Samples

Nb_2_O_5_ samples were synthesized by two different routes: namely, route 1 and route 2. For route 1, 2.0 g of the ammonium niobate (V) oxalate hydrate (general formula—H_4_[NbO­(C_2_O_4_)_2_(H_2_O)_2_]·*n*H_2_O, CBMM Brazil) were dissolved in 100 mL of water, forming a colorless solution. Hydrogen peroxide (Synth, 30% v/v) was added to this solution in a 10:1 molar ratio relative to Nb, resulting in a transparent yellow solution, indicating the formation of the niobium peroxo-complex (NPC) solution, with a pH of approximately 1.0 ± 0.5.^28,29^ Subsequently, the pH of the suspensions was adjusted using 1.0 mol L^–1^ KOH solution as needed, according to Table 1. The hydrothermal treatment for crystallization of the desired phase was carried out in a hermetically sealed reactor placed in a heating system at 120 °C for 18 h, resulting in the formation of a precipitate. The precipitate was separated by centrifugation, and then it was washed with distilled water four times to remove impurities, followed by drying in an oven at 50 °C for 12 h. For route 2, the obtained Nb_2_O_5_ nanoparticles at pH = 1.5 (±0.5) (without pH adjustment) from route 1 were dispersed in 100 mL of water. The pH was then adjusted according to the conditions outlined in Table 1, followed by a second hydrothermal treatment at 120 °C for 18 h.

**Table 1:** Nomenclature Assigned to Nb_2_O_5_ Samples Synthesized by Different Routes (1 or 2) and at Different pH Values (2, 4, and 6)

sample		pH (±0.5)
Route 1	Route 2	
Nb_2_O_5_-2	Nb_2_O_5_-AP2	2.0
Nb_2_O_5_-4	Nb_2_O_5_-AP4	4.0
Nb_2_O_5_-6	Nb_2_O_5_-AP6	6.0

### 2.2 Materials Characterization

Powder X-ray diffraction analysis was performed using a Shimadzu XRD 6000 instrument with CuKα radiation (λ = 1.5418 Å). The routine conditions used for the analysis were: 2θ scan range from 10° to 70°, with a scan speed of 2° min^–1^. The vibrational spectra in the infrared region were recorded by using a PerkinElmer FTIR spectrometer in ATR mode with a CsI detector. Raman vibrational spectra were acquired by using a HORIBA LabRAM HR Evolution spectrometer with a 532 nm excitation laser and a power of 2.5 mW. A grating of 600 lines/mm was used, and 6 accumulations were performed in the range of 75 to 4000 cm^–1^. Specific surface area measurements were carried out using nitrogen gas adsorption/desorption by the BET method, employing a Quantachrome NOVAtouch LX1 instrument. The Barret–Joyner–Halenda (BJH) numerical integration method was used to estimate the pore volume.

The XPS was performed to determine the surface compositions by using an ESCA M-Probe (Surface Science Instruments) with an Al Kα source. Data analysis was carried out with CasaXPS software (Casa Software Ltd.) after calibration to the adventitious carbon signal (284.6 eV). UV–vis diffuse reflectance spectroscopy was employed as a tool to estimate the bandgap values of the synthesized materials, performed using a Cary 5G UV–vis spectrophotometer in total reflectance mode. SEM images were obtained by using a Zeiss SUPRA 40 FEG microscope equipped with a Bruker EDS X Flash 630 M detector. TEM images were acquired using a Tecnai G2 F20 (FEI Tecnai) transmission electron microscope, operating with FEG and electron source at an acceleration voltage of 200 kV. Photoluminescence (PL) measurements were performed using a Horiba spectrofluorometer (Fluoromax-4) at room temperature, with emission spectra recorded in the 330–630 nm range under an excitation wavelength of 315 nm.

### 2.3 Photocatalytic Performance Evaluation for Organic Pollutant Degradation

The photocatalytic activity of the samples was evaluated through the photodegradation of two organic pollutants: RhB dye and Amiloride drug. For this purpose, 25.0 mg of each catalyst was added to 50 mL of RhB (5.0 mg/L) and Amiloride (10.0 mg/L). The suspensions were kept in the dark for approximately 12 h before the photocatalytic tests to evaluate possible adsorption effects. The dispersions were then stirred and exposed to ultraviolet radiation using six lamps (Philips TUV, 15 W, maximum emission centered at 254 nm, and an average irradiance intensity of 40 mW m^–2^) in a photoreactor maintained at 20 °C. The photodegradation of RhB and Amiloride was monitored at regular time intervals (0, 30, 60, 120, and 180 min) to evaluate the efficiency of the Nb_2_O_5_ samples. This was done using UV–vis spectrophotometry (Evolution 201/220, Thermo Fisher Scientific), measuring the maximum absorbance of RhB and AML at 554 and 286 nm, respectively.

The stability of the Nb_2_O_5_ photocatalyst was evaluated through four consecutive photocatalytic degradation cycles, with catalyst recovery, washing, and reuse after each cycle, while monitoring the photocatalytic efficiency throughout the repetitions.^30^ To investigate the degradation mechanism of the Nb_2_O_5_ samples, radical scavenging experiments were carried out using 50 mL of RhB solution mixed with 25 mg of the ZnO suspension. Ethylenediaminetetraacetic acid (EDTA, 10.0 μM), *tert*-butanol (*t*-BuOH, 25.0 μM), and benzoquinone (BQ, 0.5 μM) were separately added as scavengers to identify the main reactive species involved in the photocatalytic process. EDTA was used as a hole (h^+^) scavenger, tert-butanol as a hydroxyl radical (^•^OH) scavenger, and benzoquinone as a superoxide radical (O_2_^•–^) scavenger. The photocatalytic performance obtained in the presence of each scavenger was compared with that of a control experiment conducted in the absence of scavengers.^31^

### 2.4 Photocatalytic Performance Evaluation for H_2_ Evolution

Bench-scale photocatalytic tests were conducted to evaluate the efficiency of the synthesized materials in photocatalytic H_2_ production. A suspension containing 5 mg of the photocatalyst (0.5 g L^–1^) in 10 mL of an aqueous solution of methanol 20% v/v, along with 0.133 mL of a H_2_PtCl_6_ solution with a concentration of 0.376 mg mL^–1^, was prepared to achieve a platinum concentration equivalent to 1% of the Nb_2_O_5_ mass. Additionally, the effect of Pt loading on hydrogen evolution was investigated using the Nb_2_O_5_-AP2 sample with Pt contents of 0.5, 1.0, and 2.0 wt % deposited via photodeposition under the same experimental conditions. The suspension was homogenized by ultrasonication for 5 min and kept under magnetic stirring in a 16 mL borosilicate reactor under an argon atmosphere. Then, the reactor was maintained at 22 °C using a thermostatic bath and irradiated with a 300 W xenon lamp (λ > 320 nm) with an irradiance of 120 mW cm^–2^ for 30 min to promote the photodeposition of Pt nanoparticles on the Nb_2_O_5_ surface. The irradiance was measured using a Newport 1916-R Powermeter. After the deposition of Pt nanoparticles, at proper times, aliquots were collected from the reactor’s headspace and injected into a PerkinElmer Clarus 580 gas chromatograph equipped with two packed columns (Porapak N 2 mm and molecular sieve) and a thermal conductivity detector (TCD) to monitor H_2_ production. For analysis, the injector temperature was set to 120 °C, the detector temperature was set to 150 °C, and the oven temperature was ramped up from 35 to 120 °C. Argon was used as the carrier gas with a flow rate of 30 mL min^–1^. The stability experiment of the H_2_ production was performed in the same conditions for 24 h.

## 3 Results and Discussion

### 3.1 Structural and Morphological Characterization

The effect of the synthesis pH on the structure of Nb_2_O_5_ was evaluated by XRD analysis (Figure 1A,B). The X-ray diffractogram of Nb_2_O_5_·*n*H_2_O obtained by route 1 showed peaks at 2θ ≈ 22.6 (001), 27.8 (100), 46.3 (002), and 55.2 (102) being indexed to the pseudohexagonal phase (PDF: 28-317). The sample synthesized at pH 2 also presents broader peaks at approximately 2θ ≈ 12° and 2θ ≈ 26°, which are described in the literature as a transition phase between the amorphous precursor and Nb_2_O_5_ in the pseudohexagonal phase.^32^ This transition phase is related to hydrated niobium oxide (Nb_2_O_5_·*n*H_2_O).^17^ The samples synthesized by Route 1 at pH 4 (Nb_2_O_5_-4) and pH 6.0 (Nb_2_O_5_-6) showed broad-based peaks at 2θ ≈ 26–31°, related to the formation of hydrated niobium oxide (Nb_2_O_5_·*n*H_2_O).^32^ On the other hand, the samples obtained by Route 2 exhibited diffraction patterns very similar to the precursor material (Nb_2_O_5_-2); it means that the change in pH after the formation of TT-Nb_2_O_5_ obtained by Route 1 was not enough to change the crystalline phase of the materials. This behavior may be related to the high stability and low solubility of Nb_2_O_5_ in acidic and neutral solutions. Even after a second hydrothermal treatment, the crystalline structure was not changed, indicating high chemical stability of the TT-Nb_2_O_5_ structure obtained by route 1.

**Figure 1:**
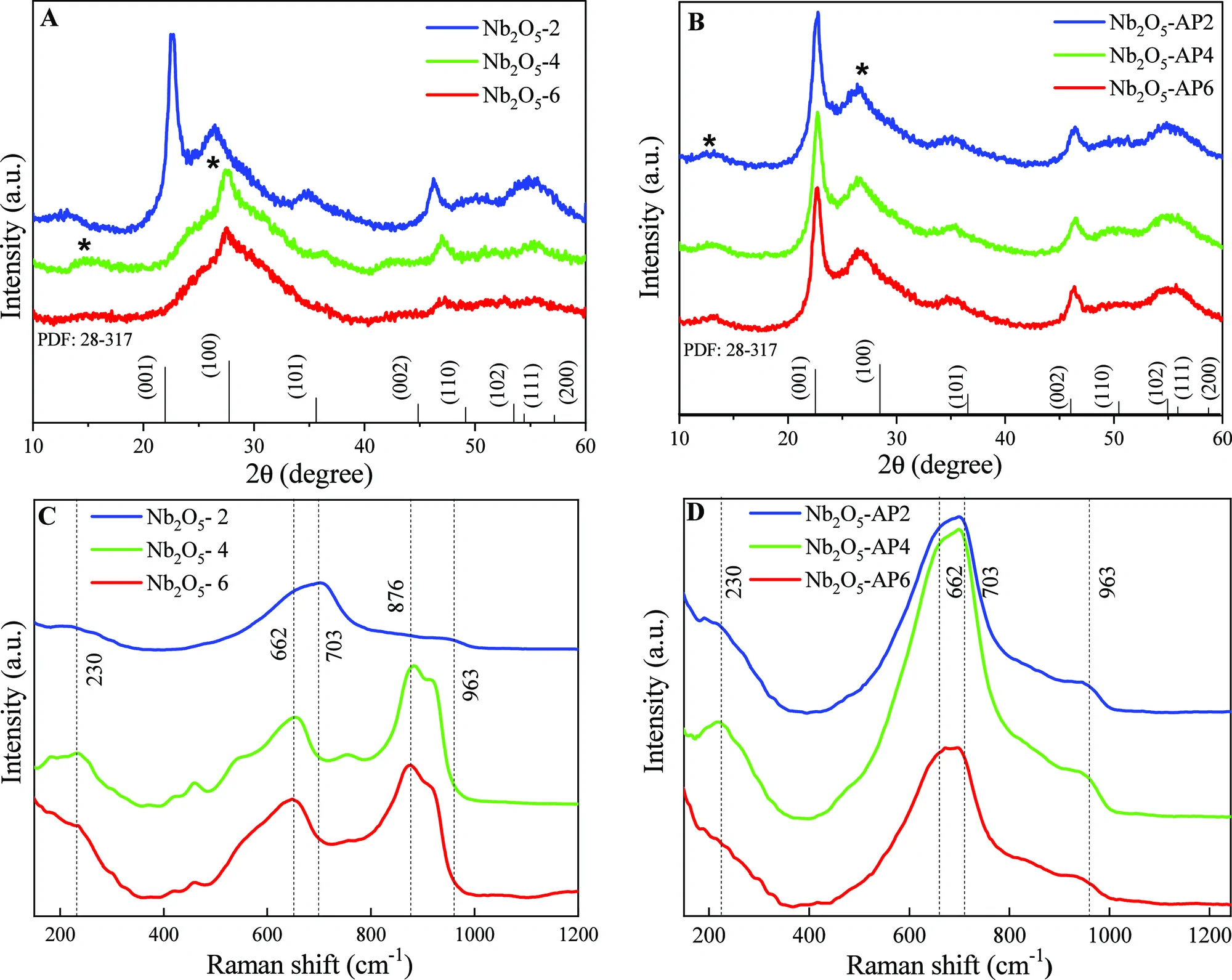
X-ray diffraction patterns for samples obtained in different pH values through (A) synthesis route 1 and (B) synthesis route 2. The simulated reference pattern corresponding to the standard Nb_2_O_5_ PDF No. 28-317 is included to facilitate comparison with the experimental diffraction peaks. Raman spectra for the samples obtained at different pH conditions at (C) route 1 and (D) route 2.

The samples were also characterized by Raman spectroscopy, as shown in Figure 1C,D. The peaks between 650 and 675 cm^–1^ are attributed to the symmetrical stretching of polyhedra Nb–O (NbO_6_^–7^ and NbO_7_^–9^, and NbO_8_^–11^), directly related to the TT-Nb_2_O_5_ phase.^33^ The peak around 652 cm^–1^ is attributed to Nb_2_O_5_·*n*H_2_O phase and the weak shoulder at 890 cm^–1^ is related to symmetric stretching of surface groups Nb=O. The vibration mode at 215 cm^–1^ is correlated to bond stretching Nb–O–Nb.^15,33^ It is possible to notice a significant change in the spectra of the samples obtained at different pH values by route 1. The samples obtained at pH 4 and 6 showed peaks attributed to the phase Nb_2_O_5_·*n*H_2_O, while the sample obtained at pH 2 showed peaks related to the phase TT-Nb_2_O_5_, in agreement with XRD results. On the other hand, the samples Nb_2_O_5_-2, Nb_2_O_5_-AP2, Nb_2_O_5_-AP4, and Nb_2_O_5_-AP6 exhibited a similar profile, which indicates that pH variation at route 2 has not caused significant changes in the materials structure. This can be explained by the low solubility of Nb_2_O_5_ in the studied pH range.

Fourier transform infrared (FTIR) spectra of the samples obtained through synthesis routes 1 and 2 can be found in Supporting Information (Figure S1). The bands observed in the low-frequency region, at 718 cm^–1^ and 500 cm^–1^, can be attributed to the stretching of the Nb=O bond and angular deformation of O–Nb–O, respectively.^34–36^ Bands associated with adsorbed water molecules are identified at 1600 cm^–1^ and 3400 cm^–1^, corresponding to the symmetric H–O–H deformation and the O–H stretching, respectively. Additionally, characteristic bands of synthesis residues are observed at approximately 1720 cm^–1^, 1404 cm^–1^, and 1200 cm^–1^. These bands are typical of the presence of carbonyl groups (C=O), which may be related to oxalate (C­(=O)_2_), and the stretching of the N–O bond, which could indicate the oxidation of ammonia to (NO_*x*_) groups. These groups are likely derived from the Nb precursor, ammonium niobate­(V) oxalate.^29,37^

XPS was employed to investigate the composition and oxidation state of the samples Nb_2_O_5_-6, Nb_2_O_5_-4, Nb_2_O_5_-AP6, and Nb_2_O_5_-AP2, with the corresponding spectra presented in Supporting Information (Figure S2). The spectra reveal the presence of Nb 3d, 1s, and 1s for all samples. The C 1s peak observed on the sample surface, ranging between 284.43 and 284.99 eV, is attributed to the carbon used for spectrum calibration.^38^ This confirms that the samples were obtained without the presence of impurities. XPS spectra of Nb 3d for the samples Nb_2_O_5_-6, Nb_2_O_5_-4, Nb_2_O_5_-AP6, and Nb_2_O_5_-AP2 revealed that the samples exhibit two prominent high-intensity bands centered at approximately 206.5 and 209.3 eV, corresponding to the splitting of the Nb^5+^ 3d orbital into Nb^5+^ 3d_5/2_.^17^ Additionally, the samples Nb_2_O_5_-6, Nb_2_O_5_-4, and Nb_2_O_5_-AP2 show a high-intensity band centered around 530.40 eV, which is associated with O 1s species in the crystalline structure of the metal oxide.^39^ These findings provide further insight into the chemical and structural characteristics of the synthesized materials.

UV–Vis DRS was employed to evaluate the optical absorption properties of the Nb_2_O_5_-based materials and their relationship with structural features (Figure 2A,B). The Nb_2_O_5_-4 and Nb_2_O_5_-6 samples exhibited absorption extending into the visible region, with an onset around 440 nm, whereas Nb_2_O_5_-2 showed an absorption edge near 380 nm, predominantly in the UV region. This red shift is associated with structural changes induced by the synthesis pH, which promotes the transition from the TT-Nb_2_O_5_ phase to hydrated Nb_2_O_5_·*n*H_2_O structures, modifying the local Nb–O environment and electronic states. In contrast, Nb_2_O_5_-AP2, Nb_2_O_5_-AP4, and Nb_2_O_5_-AP6 displayed nearly identical absorption profiles, consistent with the similar crystalline structures observed by XRD and Raman analyses.

**Figure 2:**
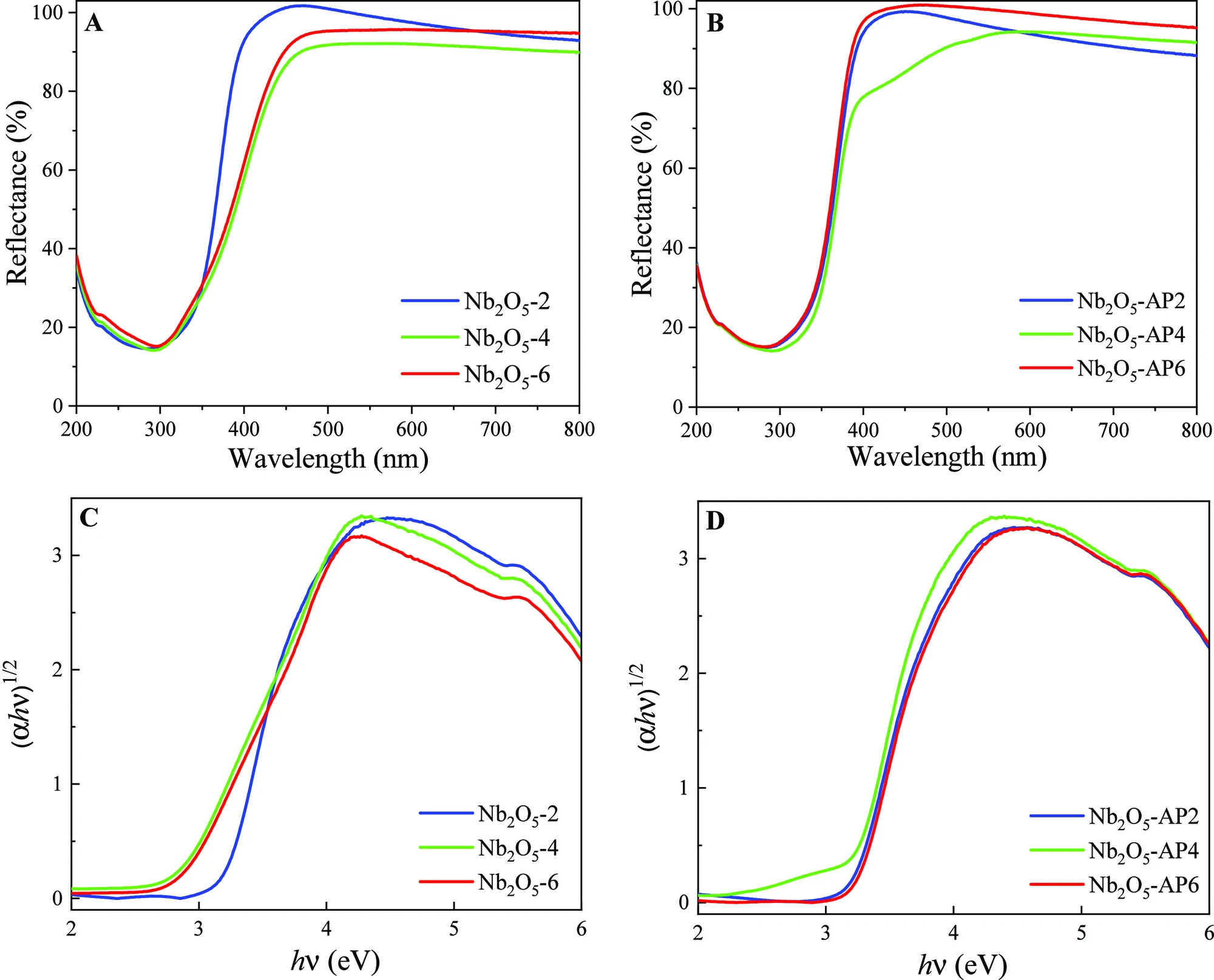
UV–vis DRS spectra of Nb_2_O_5_ samples obtained in different pH through (A) synthesis route 1 and (B) synthesis route 2. Plot of (*hv*)^2^ versus *hv* of UV–vis diffuse reflectance spectroscopy data for all synthesized samples in (C) Route 1 and (D) Route 2.

The band gap energies of the synthesized samples were determined by applying the Tauc equation to UV–vis DRS spectra, as shown in Figure 2C,D.^40^ The band gap values of the synthesized samples exhibited significant variation, ranging from 2.8 to 3.2 eV (Table 2). It was observed that samples with the TT-Nb_2_O_5_ crystalline phase (Nb_2_O_5_-2, Nb_2_O_5_-AP2, Nb_2_O_5_-AP4, and Nb_2_O_5_-AP6) displayed band gap values around 3.2 eV, consistent with previously reported data.^41^ In contrast, samples with the Nb_2_O_5_·*n*H_2_O crystalline phase (Nb_2_O_5_-4 and Nb_2_O_5_-6) exhibited lower band gap values of 2.8 eV. These results confirm that changes in the crystalline phase significantly influence the electronic properties of the materials, as evidenced by the variation in band gap energies.

**Table 2:** Band Gap Value Obtained by UV–Vis Diffuse Reflectance Spectroscopy Using the Tauc Plot

sample	band gap (eV)
Nb_2_O_5_-2	3.2
Nb_2_O_5_-4	2.8
Nb_2_O_5_-6	2.9
Nb_2_O_5_-AP2	3.2
Nb_2_O_5_-AP4	3.2
Nb_2_O_5_-AP6	3.2

The morphology of the Nb_2_O_5_ samples was examined by SEM (Figures S3 and S4). For materials synthesized via route 1, increasing the pH from 2.0 to 4.0 and 6.0 promoted the formation of plate-like particles, consistent with the lamellar stacking typical of the Nb_2_O_5_·*n*H_2_O phase commonly observed in niobate structures.^42^ This morphological evolution agrees with the phase transition identified by XRD and suggests that pH directly influences the nucleation and growth pathways. In contrast, samples obtained via route 2, consisting of the hydrothermal treatment of the Nb_2_O_5_-2 precursor at different pH values (Nb_2_O_5_-AP2, Nb_2_O_5_-AP4, and Nb_2_O_5_-AP6), exhibited agglomerates formed by spherical nanoparticles with similar sizes and an overall isotropic morphology. Such features indicate a growth process governed by more homogeneous nucleation and particle aggregation, consistent with a classical crystallization mechanism. Notably, no significant morphological variation was observed among these hydrothermally treated samples, reinforcing the structural similarity previously identified. Overall, the results demonstrate that the pH of the synthesis plays a decisive role in directing phase formation and particle morphology in route 1, whereas in route 2, the precursor structure largely governs the final morphology, leading to comparable microstructures despite pH variation.

TEM images of the Nb_2_O_5_-6 and Nb_2_O_5_-AP6 samples are presented in Figure 3. The Nb_2_O_5_-6 sample exhibits a plate-like morphology with larger dimensions, while the Nb_2_O_5_-AP6 sample displays a needle-like morphology, aggregated into nanospheres with diameters smaller than 50 nm. As observed by the SEM images. HRTEM images of the Nb_2_O_5_-6 and Nb_2_O_5_-AP6 samples are shown in Figure 4a,b, respectively. Both samples exhibit crystalline regions, as evidenced by the repetition of the lattice fringes. The Nb_2_O_5_-6 sample reveals an interlayer spacing of 0.31 nm, indicative of a stacked structure resembling a lamellar arrangement. This interlayer spacing is characteristic of the (002) plane of the HNb_3_O_8_ phase, which corresponds to a crystalline phase of Nb_2_O_5_nH_2_O,^35,43^ consistent with the XRD and Raman results. On the other hand, the Nb_2_O_5_-AP6 sample shows the interplanar spacing of 0.385 nm, which aligns well with the most prominent peak corresponding to the (001) plane of the TT-Nb_2_O_5_ phase.^28^ These findings highlight the distinct structural and morphological characteristics of the samples, which are influenced by their synthesis conditions and crystallographic phases.

**Figure 3:**
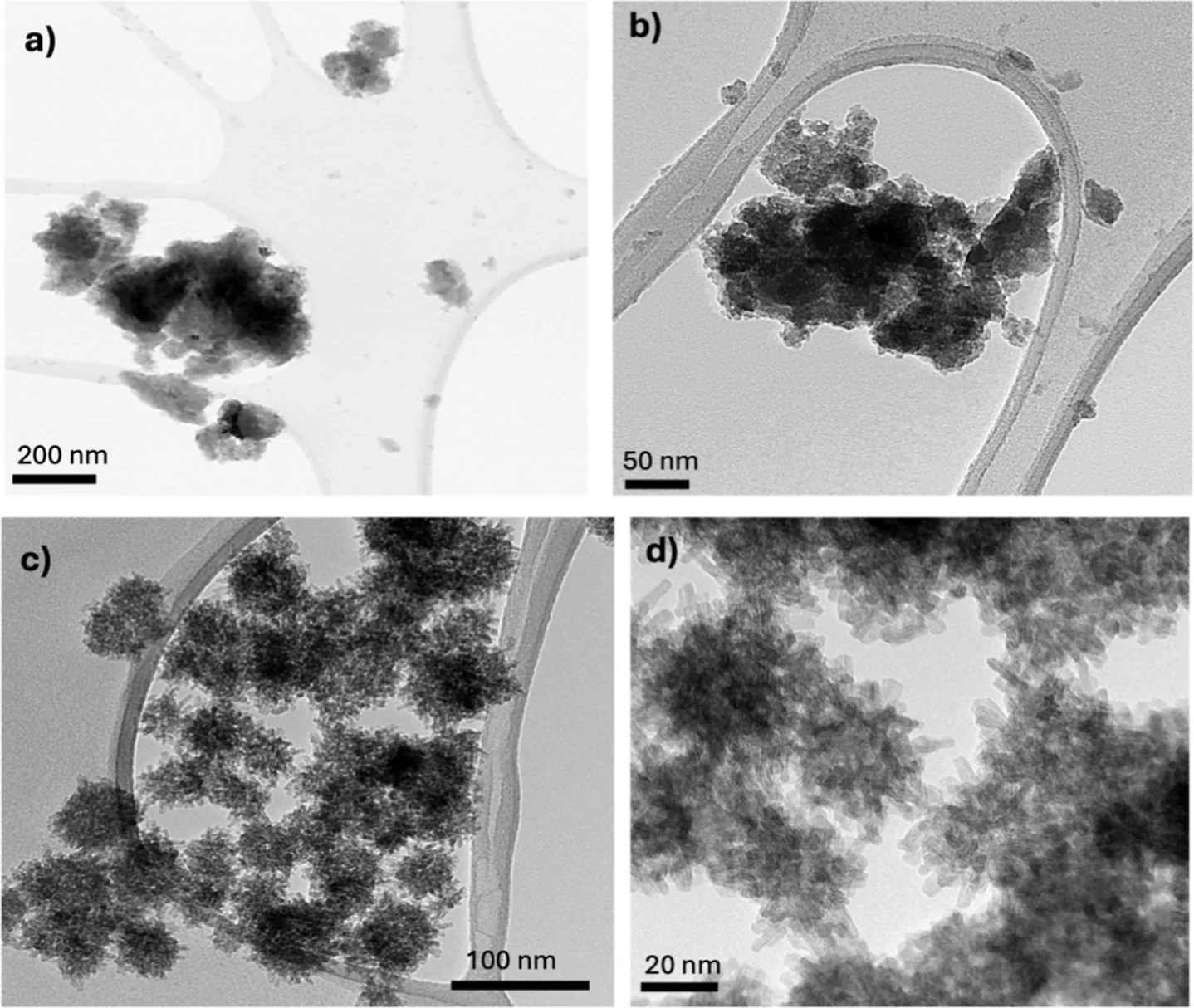
TEM images of the samples (a) and (b) Nb_2_O_5_-6 and (c) and (d) Nb_2_O_5_-AP6.

**Figure 4:**
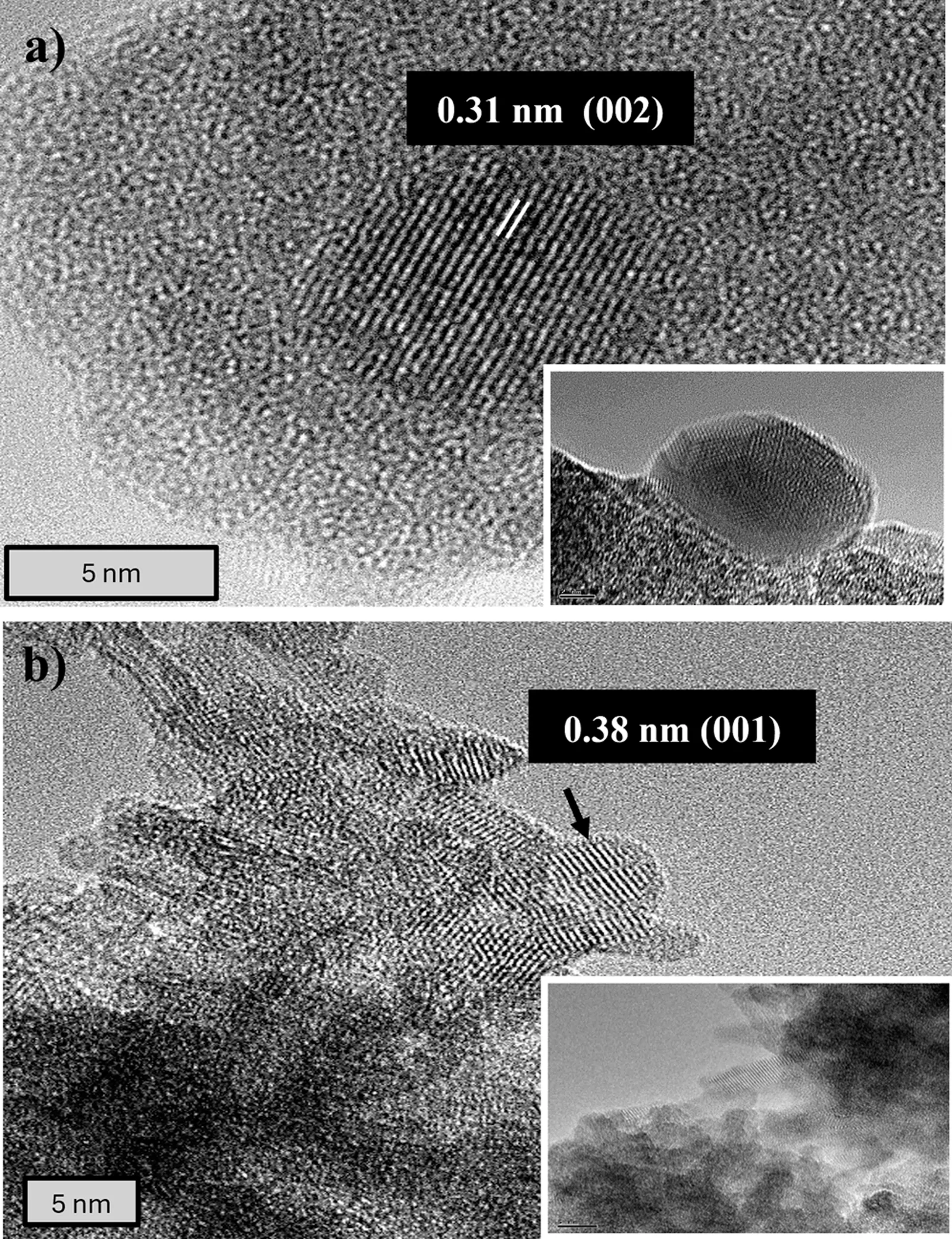
HRTEM images of the samples (a) Nb_2_O_5_-6; (b) Nb_2_O_5_-AP6.

The specific surface area of the Nb_2_O_5_-2, Nb_2_O_5_-4, Nb_2_O_5_-6, and Nb_2_O_5_-AP6 samples was analyzed by using N_2_ adsorption/desorption isotherms (Table 3). As a result of the treatment and the increase in pH, a significant enhancement in the specific surface area was observed. This increase can be attributed to changes in the nucleation and growth mechanisms of the particles, as evidenced by SEM images. Since photocatalytic reactions occur on the surface of photocatalysts, the specific surface area directly influences the photoactivity of the material. Additionally, the specific surface area is directly related to the adsorption capacity of the photocatalyst.^12,13^ A larger surface area is inherently advantageous for the generation of active sites on a larger scale.^12^ These findings underscore the importance of optimizing synthesis conditions to enhance the textural properties of photocatalysts, thereby improving their performance in photocatalytic applications.

**Table 3:** Textural Parameters for Nb_2_O_5_-2, Nb_2_O_5_-4, Nb_2_O_5_-AP2, and Nb_2_O_5_-AP6

sample	specific surface area (m^2^ g^–1^)	average pore size (nm)	total pore volume (cm^3^ g^–1^)
Nb_2_O_5_-2	185.0	6.0	0.6
Nb_2_O_5_-4	172.0	2.6	0.3
Nb_2_O_5_-6	207.0	1.6	0.2
Nb_2_O_5_-AP6	224.0	4.4	0.5

### 3.2 Photocatalytic Performance Evaluation

The photocatalytic performance of the synthesized samples was initially evaluated through photodegradation assays of RhB, monitoring the dye concentration during exposure under UV radiation, and the pseudo-first-order kinetics evaluation (Figure 5). RhB underwent partial degradation via direct photolysis, corresponding to approximately 15% removal under UV irradiation in the absence of a catalyst. Therefore, all synthesized samples exhibit photocatalytic activity in the degradation of RhB, as they induced dye degradation exceeding the 15% threshold achieved by direct photolysis. The results further reveal that samples synthesized at pH 6, regardless of the synthesis route, demonstrated superior photocatalytic activity compared to samples obtained at lower pH values. Notably, these samples outperformed commercial Nb_2_O_5_, highlighting the influence of synthesis conditions on the photocatalytic efficiency.

**Figure 5:**
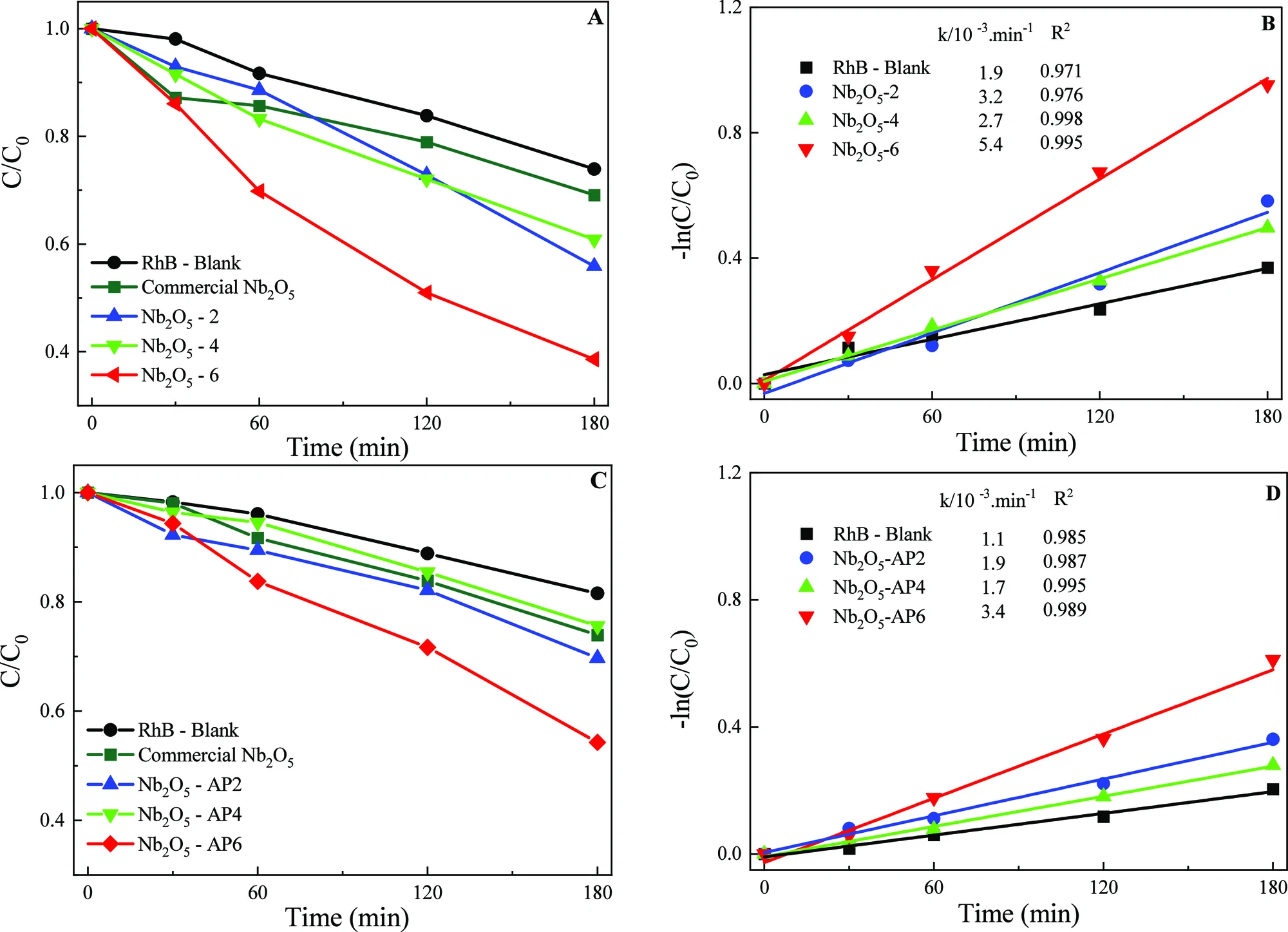
(A) Degradation curve of the Rhodamine B dye as a function of the exposure time under UV radiation catalyzed by the Nb_2_O_5_ samples obtained by route 1; (B) pseudo-first order kinetics of the samples synthesized by route 1; (C) degradation curve of the Rhodamine B dye as a function of the exposure time under UV radiation catalyzed by the Nb_2_O_5_ samples obtained by route 2; (D) pseudo-first order kinetics of the samples synthesized by route 2.

The enhanced performance is likely attributed to the increased surface area observed in samples synthesized at higher pH values, particularly those produced via route 2. Interestingly, while the samples synthesized via route 2 (Nb_2_O_5_-AP2, Nb_2_O_5_-AP4, and Nb_2_O_5_-AP6) exhibited similar structural and electronic properties, their photocatalytic performance varied, suggesting that morphological differences and surface area are the primary factors influencing their activity.^44,45^ This observation underscores the critical role of surface characteristics in determining photocatalytic efficiency, even when the electronic and structural properties remain similar. Therefore, both the synthesis route and pH conditions significantly affect the photocatalytic performance of the synthesized materials. These insights contribute to a broader understanding of material design for advanced photocatalytic applications, particularly in environmental remediation processes.

In order to assess the versatility of the synthesized samples to promote the degradation of the different kinds of organic pollutants, the AML drug was employed for photodegradation tests. Amiloride is a pharmaceutical compound widely used as a diuretic and in the treatment of hypertension and heart failure. Due to its persistence and biological activity, residues of amiloride in wastewater pose environmental and ecological risks, particularly to aquatic systems.^46,47^ Figure 6 exhibits the photocatalytic degradation curves and the pseudo-first-order kinetics of AML catalyzed by the Nb_2_O_5_ samples. The samples synthesized via route 1 did not exhibit significant differences in photoactivity with respect to pH variation. This behavior may be attributed to the fact that the samples obtained at pH 4 and 6 adsorbed approximately 49% and 75% AML, respectively, prior to the commencement of the photocatalysis process. Therefore, the observed removal of AML for these samples is more closely associated with the adsorption process, rather than photocatalysis. Conversely, the sample synthesized at pH 2 demonstrated superior performance in the photodegradation of AML. For the sample obtained by route 2 at pH 6, the rate constant of the AML degradation reaction was slightly higher than that of the samples obtained at pH 2 and 4. Since Nb_2_O_5_-2 did not exhibit significant adsorption of AML, it suggests that the TT-Nb_2_O_5_ phase was more efficient in the degradation of this pollutant compared to the Nb_2_O_5_·*n*H_2_O phase obtained at pH 4 and 6. This behavior is corroborated by the performance evaluation of the samples synthesized via route 2, where no significant adsorption of AML was observed, and all samples effectively degraded the pollutant. This efficiency can be attributed to the fact that all route 2 samples were obtained in the TT-Nb_2_O_5_ phase. These findings are particularly relevant, as they demonstrate that samples in the TT-Nb_2_O_5_ phase exhibit high efficiency in the degradation of organic pollutants with different physicochemical properties.

**Figure 6:**
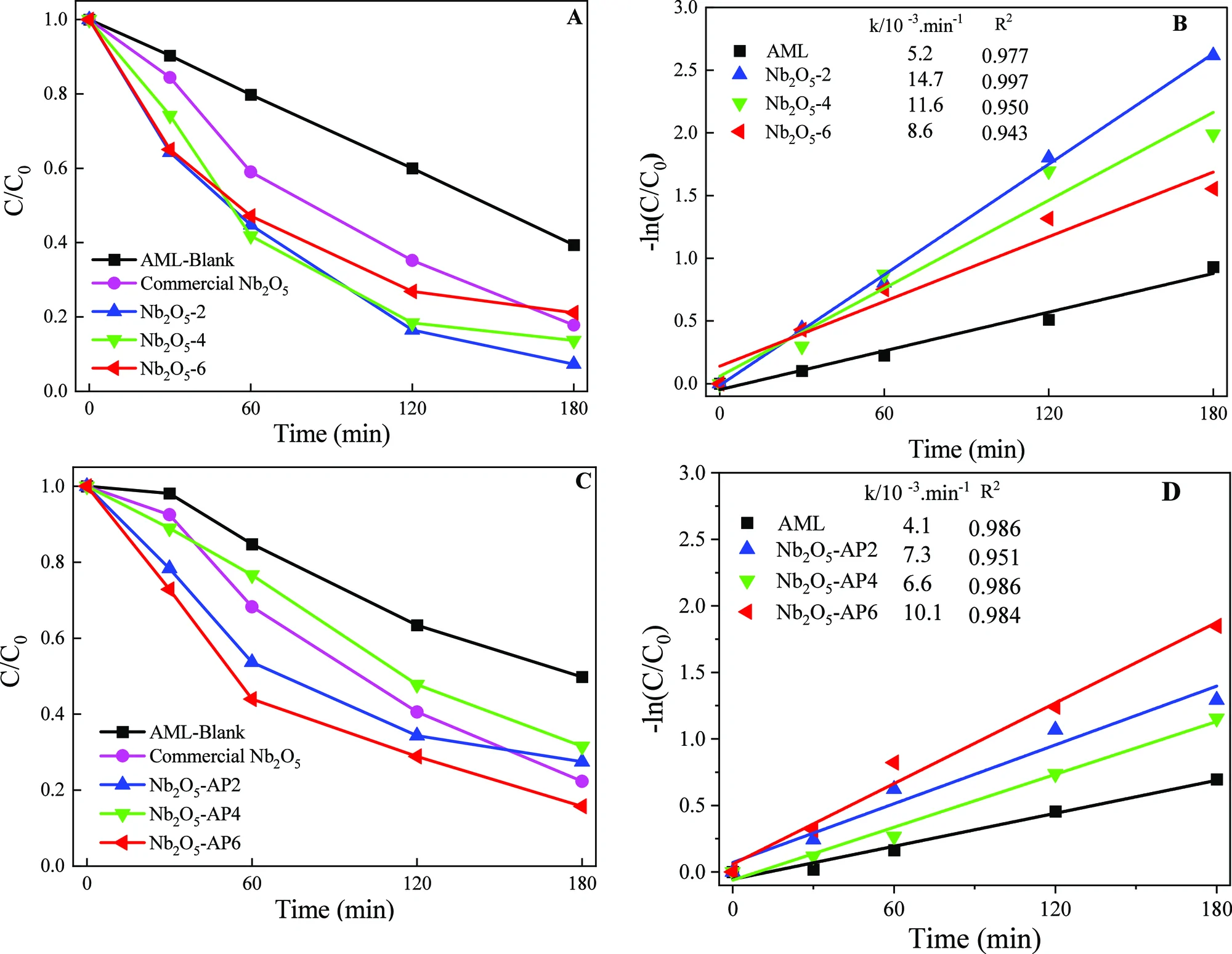
(A) Degradation curve of AML as a function of the exposure time under UV radiation catalyzed by the Nb_2_O_5_ samples obtained by route 1; (B) pseudo-first order kinetics of the samples synthesized by route 1; (C) degradation curve of the AML as a function of the exposure time under UV radiation catalyzed by the Nb_2_O_5_ samples obtained by route 2; (D) pseudo-first order kinetics of the samples synthesized by route 2.

Samples synthesized via Route 1 exhibited negligible variation in degradation rates, resulting in no significant changes in the half-life of the drug. This suggests that the catalytic properties of the materials produced through this route were insufficient to substantially alter the degradation kinetics. In contrast, samples obtained from the TT phase (Route 2) demonstrated notable catalytic activity, significantly enhancing the degradation of the drug. These findings highlight the critical role of synthesis routes in determining the catalytic performance of materials. The enhanced degradation efficiency observed in Route 2 samples, especially under mildly acidic conditions (pH 6), underscores the potential of the TT phase as an effective catalyst for drug degradation processes.

The recyclability and stability of Nb_2_O_5_-6 and Nb_2_O_5_-AP6 photocatalysts were evaluated through four consecutive RhB photodegradation cycles in order to assess their structural robustness and long-term applicability. As shown in Figure 7A,B, both samples maintained nearly constant photocatalytic performance throughout all reuse cycles, indicating excellent stability under UV irradiation conditions with performance above 80%. The absence of significant loss in degradation efficiency suggests that neither catalyst underwent substantial surface deactivation, photocorrosion, or structural deterioration during the photocatalytic process.^48^ This behavior highlights the intrinsic chemical stability of Nb_2_O_5_-based materials, which is commonly associated with the strong Nb–O framework and high resistance to oxidative environments.^49,50^ The preservation of activity after repeated cycles also indicates that the active surface sites responsible for the generation of reactive oxygen species remained accessible and functional.

**Figure 7:**
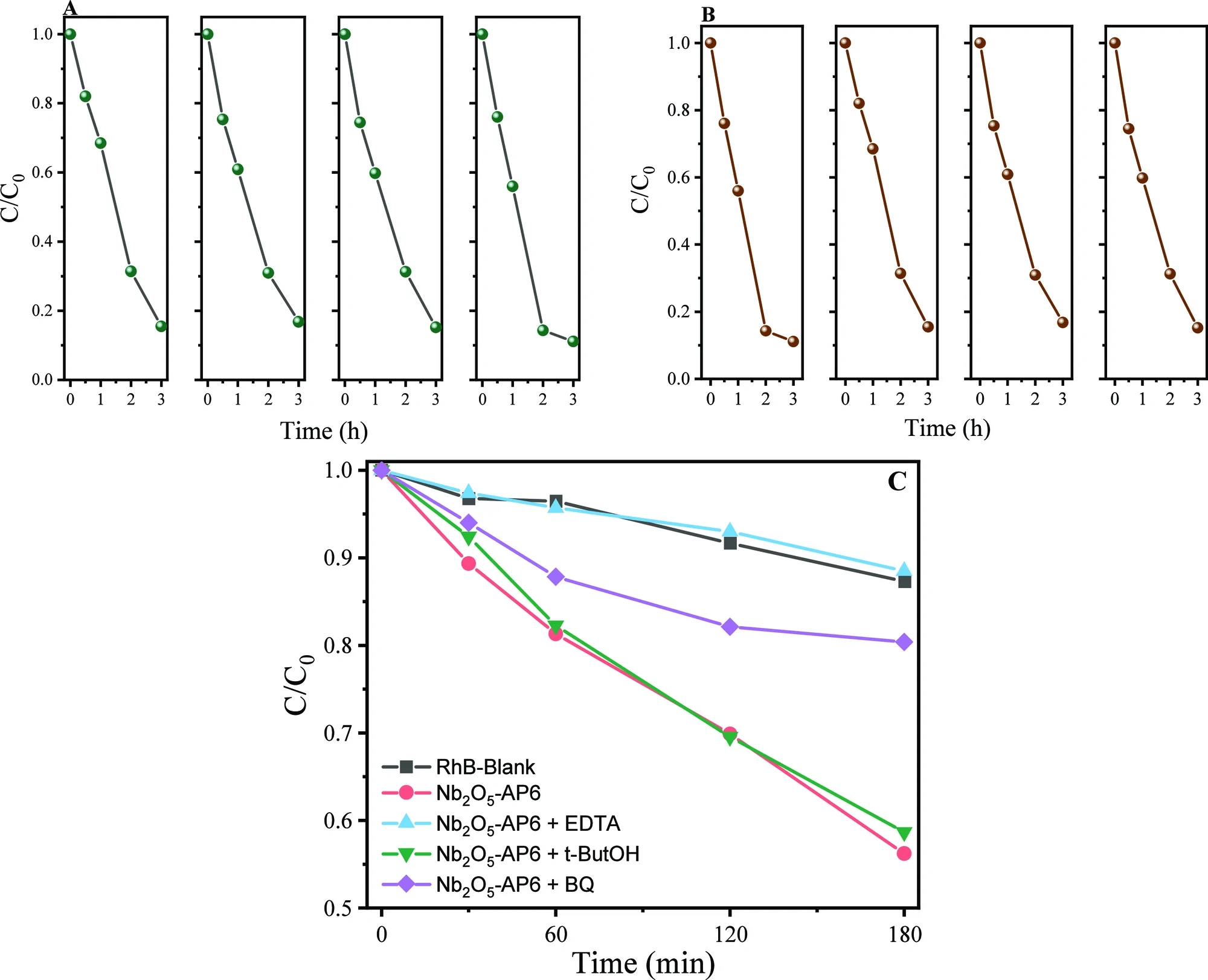
Reusability and stability performance of (A) Nb_2_O_5_-6 and (B) Nb_2_O_5_-AP6 photocatalysts over four consecutive photocatalytic cycles for Rhodamine B degradation, illustrating the retention of photocatalytic activity after repeated reuse. (C) Kinetic study of RhB dye photodegradation over the Nb_2_O_5_-AP6 photocatalyst in the presence of radical scavengers (EDTA, benzoquinone, and tert-butanol), elucidating the role of photogenerated h^+^, O_2_^•–^, and ^•^OH in the photocatalytic reaction mechanism.

The photocatalytic degradation mechanism of RhB over Nb_2_O_5_-AP6 was investigated by using radical scavenger experiments with EDTA, BQ, and *t*-ButOH, acting as h^+^, O_2_^•–^, and ^•^OH scavengers, respectively. As shown in Figure 7C, the addition of EDTA resulted in a complete inhibition of RhB degradation, producing a kinetic profile similar to that of the dye without a photocatalyst, clearly indicating that photogenerated h^+^ are the main active species in the reaction.^48^ The presence of BQ also caused a strong suppression of the degradation rate, demonstrating the significant contribution of O_2_^•–^ to the photocatalytic process. In contrast, the addition of t-ButOH produced negligible changes in degradation kinetics, indicating that ^•^OH plays an insignificant role under the studied conditions.^48,51^ These results confirm that the photocatalytic oxidation of RhB over Nb_2_O_5_-AP6 is predominantly driven by hole-mediated oxidation, followed by a secondary contribution from O_2_^•–^ species, while ^•^OH radicals have minimal participation in the reaction pathway.^52^

### 3.3 Hydrogen Production Assays

The photocatalytic activity of Nb_2_O_5_ samples synthesized via both routes was evaluated for hydrogen production under UV–vis irradiation (λ > 350 nm) using a 20% v/v aqueous methanol solution as a hole scavenger, i.e., sacrificial reagent. Pt was photodeposited on the surface of the samples to act as a cocatalyst, aiming to increase the photocatalytic efficiency. The hydrogen production kinetics are illustrated in Figure 8a, while the concentration of H_2_ per gram of photocatalyst after 8 h of reaction is summarized in Table 4.

**Figure 8:**
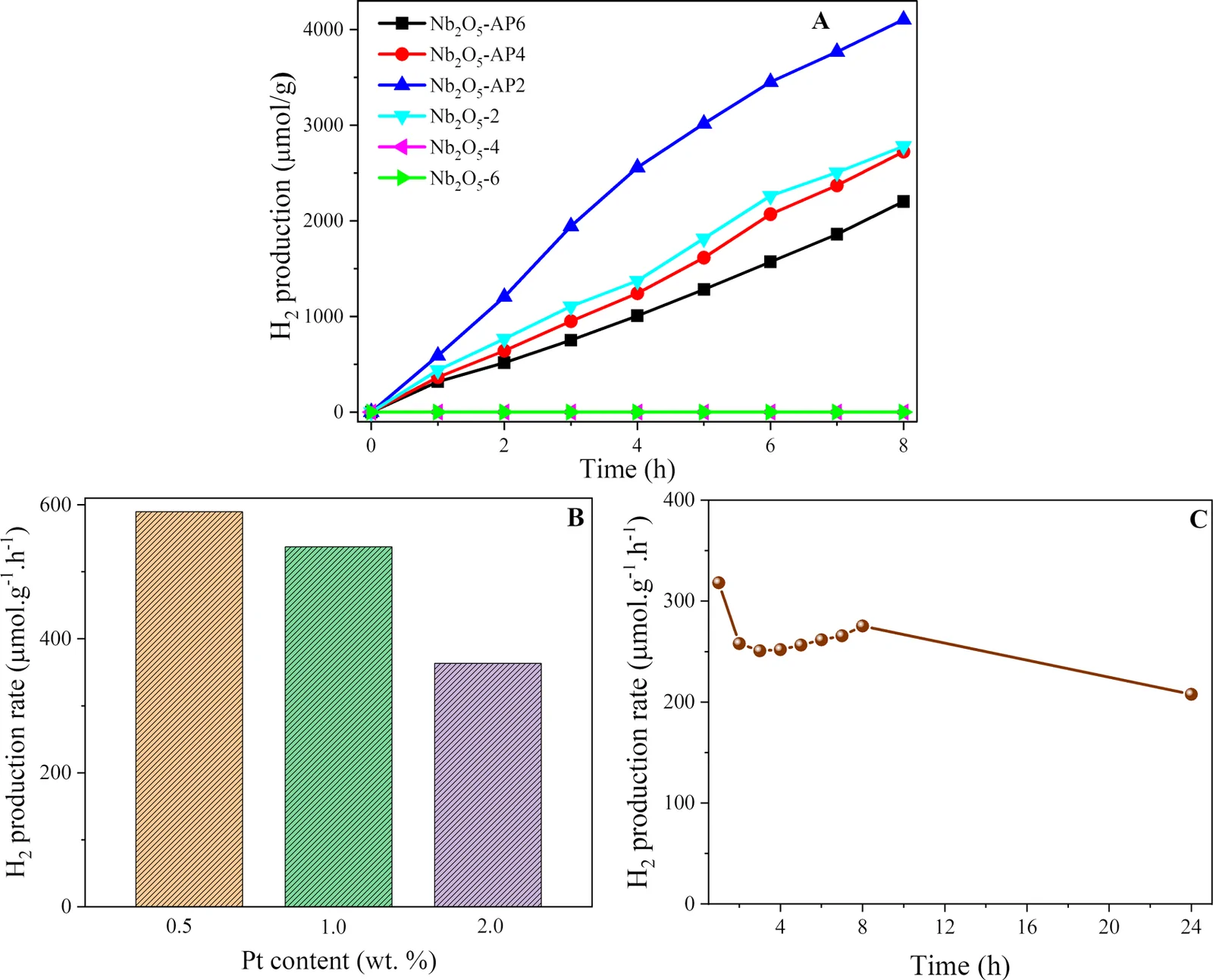
(A) H_2_ production as a function of UV–vis irradiation time over Nb_2_O_5_/Pt (1 wt %) catalysts using methanol as a sacrificial reagent. (B) Hydrogen production rate after 2 h reaction for Nb_2_O_5_-AP2 catalysts containing different Pt loadings (0.5–2 wt %). (C) Photocatalytic stability evaluation of the H_2_ production rate over 24 h using Nb_2_O_5_-AP6/Pt (1 wt %) as a catalyst.

**Table 4:** H_2_ Production After 8 h of UV–Vis Exposure Catalyzed by Nb_2_O_5_/Pt (1 wt %) Samples Using Methanol as Sacrificial Reagent

sample	H_2_ concentration (μmol g^–1^)
Nb_2_O_5_-2	2780
Nb_2_O_5_-4	0.0
Nb_2_O_5_-6	0.0
Nb_2_O_5_-AP2	4105
Nb_2_O_5_-AP4	2723
Nb_2_O_5_-AP6	1678

The photodeposition of platinum on the semiconductor surface plays a pivotal role in the photogeneration of H_2_ in the presence of a sacrificial reagent, such as methanol. Platinum nanoparticles facilitate a more efficient charge separation, significantly extending the lifetime of the electron–hole (e–/h+) pairs. This enhancement promotes the evolution of hydrogen from water. Furthermore, platinum reduces the activation energy of the overall process, contributing to a more effective generation of H_2_.^53,54^ The results demonstrate that the incorporation of Pt nanoparticles on the Nb_2_O_5_ surface markedly improves the photocatalytic performance, highlighting the importance of cocatalysts in optimizing hydrogen production.

Nb_2_O_5_-AP2 exhibited the highest photocatalytic response, producing 4104.5 μmol g^–1^ of H_2_. In contrast, Nb_2_O_5_-4 and Nb_2_O_5_-6 showed no measurable photocatalytic hydrogen production. This may be attributed to their low band gap values (2.8–2.9 eV), which likely favor charge carrier recombination due to the low reduction potential of the conduction band. Thus, these results highlight the critical influence of synthesis conditions on the photocatalytic efficiency of the Nb_2_O_5_-based materials. The second hydrothermal treatment of the Nb_2_O_5_-2 sample at pH 2 resulted in a remarkable enhancement of its photocatalytic activity. This improvement is attributed to the effective removal of surface groups derived from synthesis residues, which likely hindered the charge transfer processes in the untreated sample. However, the second hydrothermal treatments at pH 4 and 6 did not yield significant differences in photocatalytic performance compared to the untreated Nb_2_O_5_-2 sample. This suggests that the pH of the post-treatment plays a crucial role in optimizing the material’s surface properties and, consequently, its photocatalytic activity.

Photoluminescence (PL) spectra (Figure S5) were analyzed to further investigate the charge carrier recombination behavior of the samples. The Nb_2_O_5_-AP2 material exhibits a slightly lower emission intensity compared to those of the other samples, suggesting a modest reduction in radiative electron–hole recombination. However, the differences in PL intensity are relatively small, indicating that recombination suppression alone does not fully account for the superior photocatalytic performance observed. Therefore, the enhanced H_2_ production activity of Nb_2_O_5_-AP2 is likely associated with a combination of factors, including improved surface properties and charge-transfer processes promoted by the second hydrothermal treatment, rather than sole changes in electronic recombination dynamics.

Interestingly, the trends observed in hydrogen production reactions differed from those in organic pollutant photodegradation, indicating that distinct material properties govern the performance in these two processes. This underscores the importance of tailoring synthesis parameters, such as pH, to optimize materials for specific applications. The photocatalytic results for hydrogen production confirmed that samples with a nanoneedle morphology and the TT-Nb_2_O_5_ crystalline phase exhibited superior performance across all photocatalytic tests compared to that of Nb_2_O_5_·*n*H_2_O materials. Several factors may limit the photocatalytic efficiency of Nb_2_O_5_·*n*H_2_O, including low charge separation efficiency after bandgap excitation, inefficient charge transfer to platinum nanoparticles, limitations in the surface reduction reaction or structural defects that can increase charge carrier recombination rates, thereby reducing photocatalytic efficiency.^55,56^

Furthermore, the Nb_2_O_5_·*n*H_2_O phase, characterized by a narrower bandgap, may exhibit reduced redox potentials in its conduction and valence bands, further limiting its photocatalytic activity. In a related study, Zhou et al. reported the synthesis of monoclinic Nb_2_O_5_ superstructures using Sn_2_Nb_2_O_7_ nanoparticles as precursors. The light absorption properties of Nb_2_O_5_ nanorods were compared to those of commercial Nb_2_O_5_ powders, revealing a significant blue shift in the absorption edge for the nanorods, likely due to their nanometric size. Photocatalytic hydrogen production was evaluated using 0.5 wt % Pt as a cocatalyst in a 25% aqueous methanol solution under irradiation from a 500 W high-pressure mercury lamp. The Nb_2_O_5_ nanorods demonstrated a hydrogen evolution rate of 91 μmol h^–1^, while the commercial sample exhibited a rate of only 0.6 μmol h^–1^, approximately 150 times lower. This further emphasizes the superior performance of nanostructured Nb_2_O_5_ materials in photocatalytic applications. These findings collectively highlight the importance of morphology, crystallinity, and surface properties in determining the photocatalytic performance of the Nb_2_O_5_-based materials.

Figure 8b presents the hydrogen production rate of Nb_2_O_5_-AP2 catalysts containing different Pt loadings (0.5–2 wt %). The results reveal a clear dependence of the photocatalytic performance on the amount of Pt deposited on the Nb_2_O_5_ surface. The catalyst containing 0.5 wt % Pt exhibited the highest H_2_ production rate, indicating that relatively low cocatalyst loading is sufficient to promote efficient charge separation and hydrogen evolution. Increasing the Pt loading to 1 wt % resulted in a slight decrease in photocatalytic activity, while further increasing the loading to 2 wt % led to a significant reduction in performance, approximately 40% lower than that obtained for the 0.5 wt % Pt sample.

This behavior is consistent with reports in the literature where an optimal noble metal loading is commonly observed for photocatalytic hydrogen evolution systems. Small amounts of Pt act as effective electron sinks, facilitating charge separation and lowering the activation energy for proton reduction.^57^ However, excessive Pt deposition may partially cover the semiconductor surface, blocking active sites and reducing light absorption by Nb_2_O_5_ due to a shielding effect.^58^ In addition, high metal loadings can promote electron recombination at metal clusters or reduce the semiconductor–solution interfacial area, ultimately decreasing photocatalytic efficiency.^59^

The photocatalytic stability of the Nb_2_O_5_-AP6/Pt (1 wt %) catalyst was evaluated over 24 h of continuous irradiation (Figure 7c). During the first 8 h of the reaction, the hydrogen production rate remained essentially constant, indicating stable catalytic activity and efficient charge transfer processes. After prolonged irradiation (24 h), a slight decrease in the reaction rate was observed; however, H_2_ evolution continued, demonstrating that the catalyst preserved its photocatalytic functionality. This mild deactivation after extended operation has also been described in the literature and is generally attributed to factors such as partial surface restructuring, accumulation of reaction intermediates, or minor aggregation of Pt nanoparticles during long-term irradiation.^60^

Importantly, the absence of a drastic decline in activity confirms the high stability of the Nb_2_O_5_-based photocatalyst and the strong metal-support interaction between Pt and the TT-Nb_2_O_5_ structure. Overall, the results demonstrate that optimizing Pt loading is crucial to maximizing photocatalytic efficiency, while the Nb_2_O_5_-AP6/Pt system exhibits good operational stability, maintaining effective hydrogen production even after extended reaction times.

## 4 Conclusions

This study demonstrates that the synthesis pH is a key parameter governing the phase composition, morphology, surface chemistry, and photoinduced charge-carrier dynamics of Nb_2_O_5_, ultimately determining its photocatalytic performance in both environmental remediation and hydrogen production. For materials prepared via Route 1, pH 2 favored the formation of the pseudohexagonal TT-Nb_2_O_5_ phase with nanoneedle morphology, whereas pH 4 and 6 led to the formation of hydrated Nb_2_O_5_·*n*H_2_O with lamellar features. In contrast, samples obtained via Route 2 preserved the TT-Nb_2_O_5_ phase regardless of postsynthesis pH adjustment, highlighting the structural stability and low solubility of this phase under hydrothermal conditions. These phase-dependent structural differences directly influenced optical absorption, band gap energies, and charge-transfer properties.

All samples were active in the degradation of RhB dye and AML drug, where Nb_2_O_5_·*n*H_2_O materials exhibited strong adsorption capacity, contributing significantly to pollutant removal, while TT-Nb_2_O_5_ samples showed superior intrinsic photocatalytic efficiency. Radical scavenger experiments confirmed that RhB degradation over Nb_2_O_5_-AP6 is predominantly hole-driven, with a secondary contribution from O_2_^–•^ and negligible participation of ^•^OH. Recycling tests demonstrated high structural stability and sustained activity over four consecutive cycles, confirming the robustness of Nb_2_O_5_ under photocatalytic conditions.

In hydrogen evolution reactions, activity was strictly associated with the TT-Nb_2_O_5_ phase, emphasizing the importance of the electronic structure and charge mobility. Nb_2_O_5_-AP2 achieved the highest H_2_ production (4104.5 μmol g^–1^ after 8 h). The Pt loading study revealed an optimal cocatalyst content (0.5 wt %), where efficient electron trapping maximized hydrogen evolution, while higher loadings induced surface shielding and partial recombination losses. Photoluminescence analyses showed only subtle differences in emission intensity, indicating that although recombination suppression contributes to performance enhancement, the superior activity of Nb_2_O_5_ -AP2 arises from a synergistic combination of improved surface cleanliness, optimized charge transfer, and favorable metal-support interactions. Long-term assays confirmed stable H_2_ evolution over 24 h with only minor activity decay, demonstrating good operational durability.

Overall, the results establish clear correlations among synthesis conditions, crystalline phase, surface properties, and application-specific photocatalytic behavior. While Nb_2_O_5_·*n*H_2_O is advantageous for adsorption-assisted pollutant removal, TT-Nb_2_O_5_ is more suitable for redox-demanding reactions such as hydrogen evolution. These findings provide mechanistic and structural insights for the rational design of Nb_2_O_5_-based photocatalysts tailored for integrated environmental and energy applications.

## Supplementary Material



## Notes

Fourier transform infrared spectroscopy (FTIR) of Nb_2_O_5_ samples synthesized by different routes; XPS analysis of Nb_2_O_5_-6, Nb_2_O_5_-4, Nb_2_O_5_-AP6, and Nb_2_O_5_-AP2; SEM images of Nb_2_O_5_-2, Nb_2_O_5_-4, and Nb_2_O_5_-6; SEM images of Nb_2_O_5_-AP2, Nb_2_O_5_-AP4, and Nb_2_O_5_-AP6; and photoluminescence emission spectra of Nb_2_O_5_ samples using excitation at 340 nm (PDF)
