# Supplementary material for: pH-Controlled Synthesis of Nb2O5 Nanoparticles with Enhanced Performance for Photodegradation of Organic Pollutants and H2 Production
Source: ACS Phys Chem Au. 2026 Mar 18;6(5):1018–30. doi: 10.1021/acsphyschemau.5c00132 (PMC13614168; doi:10.1021/acsphyschemau.5c00132)
Supplement: Supplementary file 1 [file pg5c00132_si_001.pdf]

SUPPORTING INFORMATION

pH-Controlled synthesis of Nb_2O_5 nanoparticles with enhanced performance for photodegradation of organic pollutants and H_2 production

Carolina X. J. Silva[1], *Marcela Piassi Bernardo*[1], Ivan José Silva Jr.[1], *Sheila Cristina Canobre*[1], *Osmando F. Lopes*[1]*

[1] Institute of Chemistry, Federal University of Uberlândia (IQUFU), Av. João Naves de Ávila , 2121, Santa Monica, 38400-902 Uberlândia-MG, Brazil

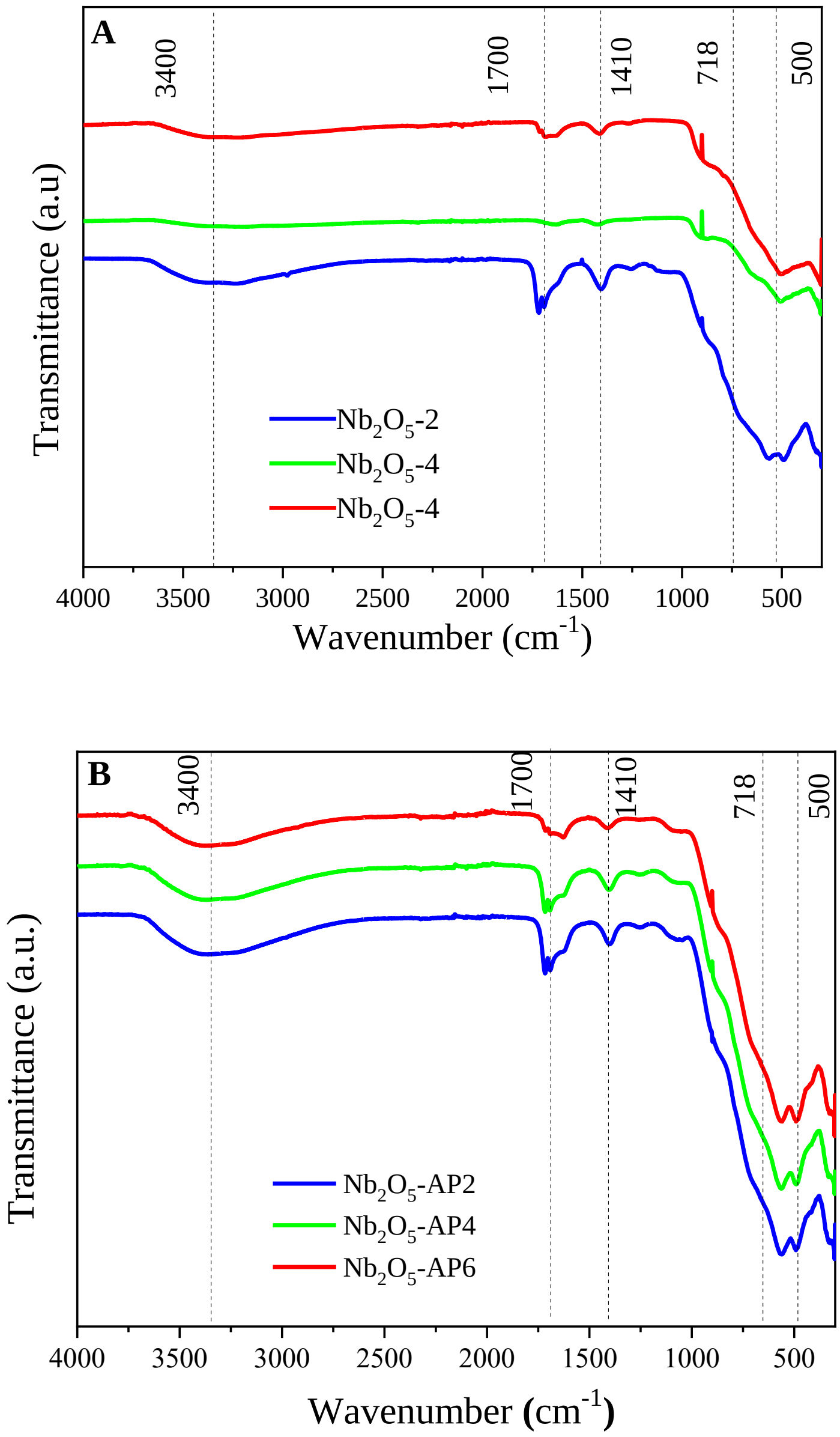

Figure S1. Fourier Transform Infrared Spectroscopy of Nb_2O_5 samples synthesized through a) route 1 and b) route 2

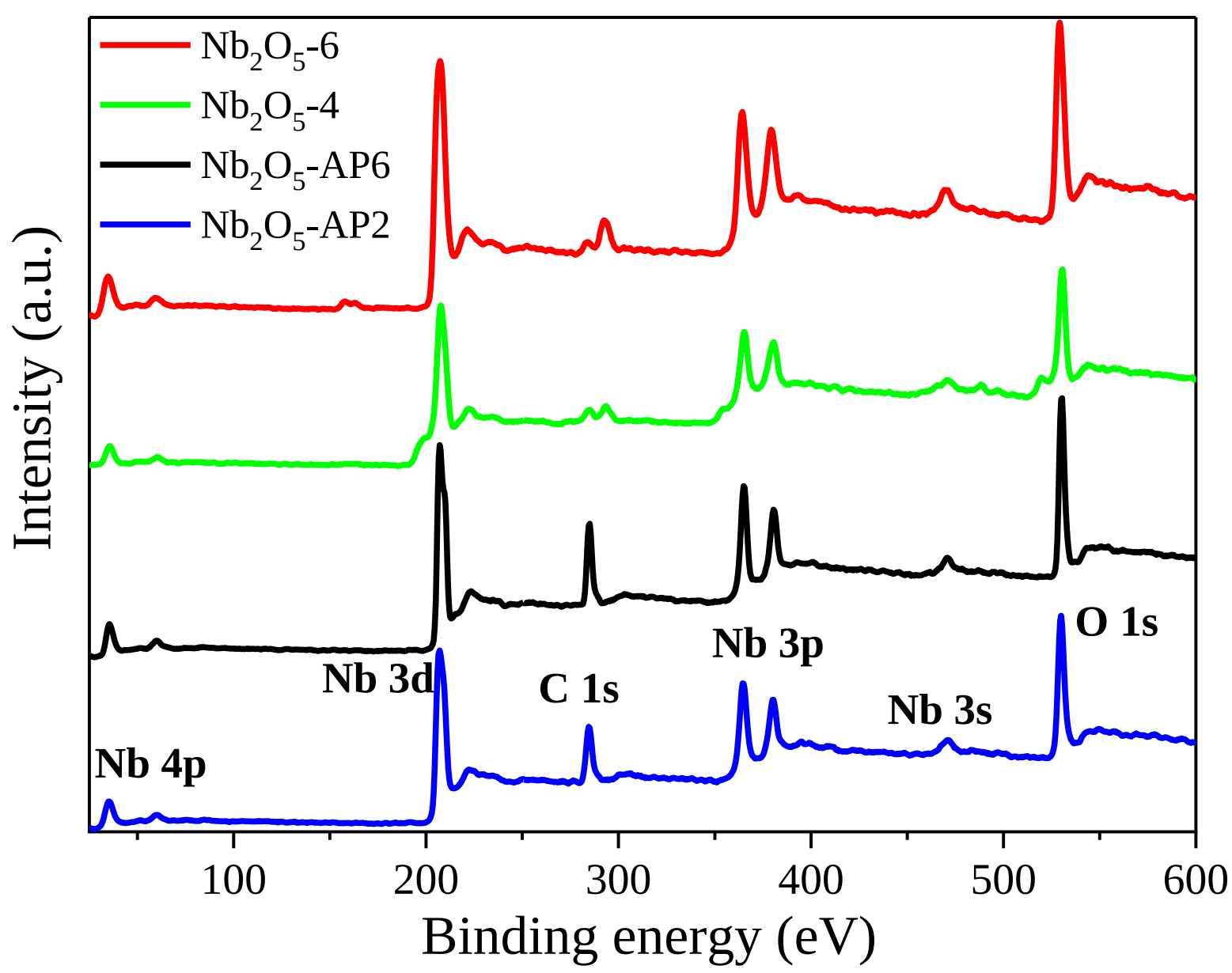

Figure S2. X-ray photoelectron spectroscopy of samples Nb_2O_5-6, Nb_2O_5-4, Nb_2O_5-AP6 and Nb_2O_5-AP2.

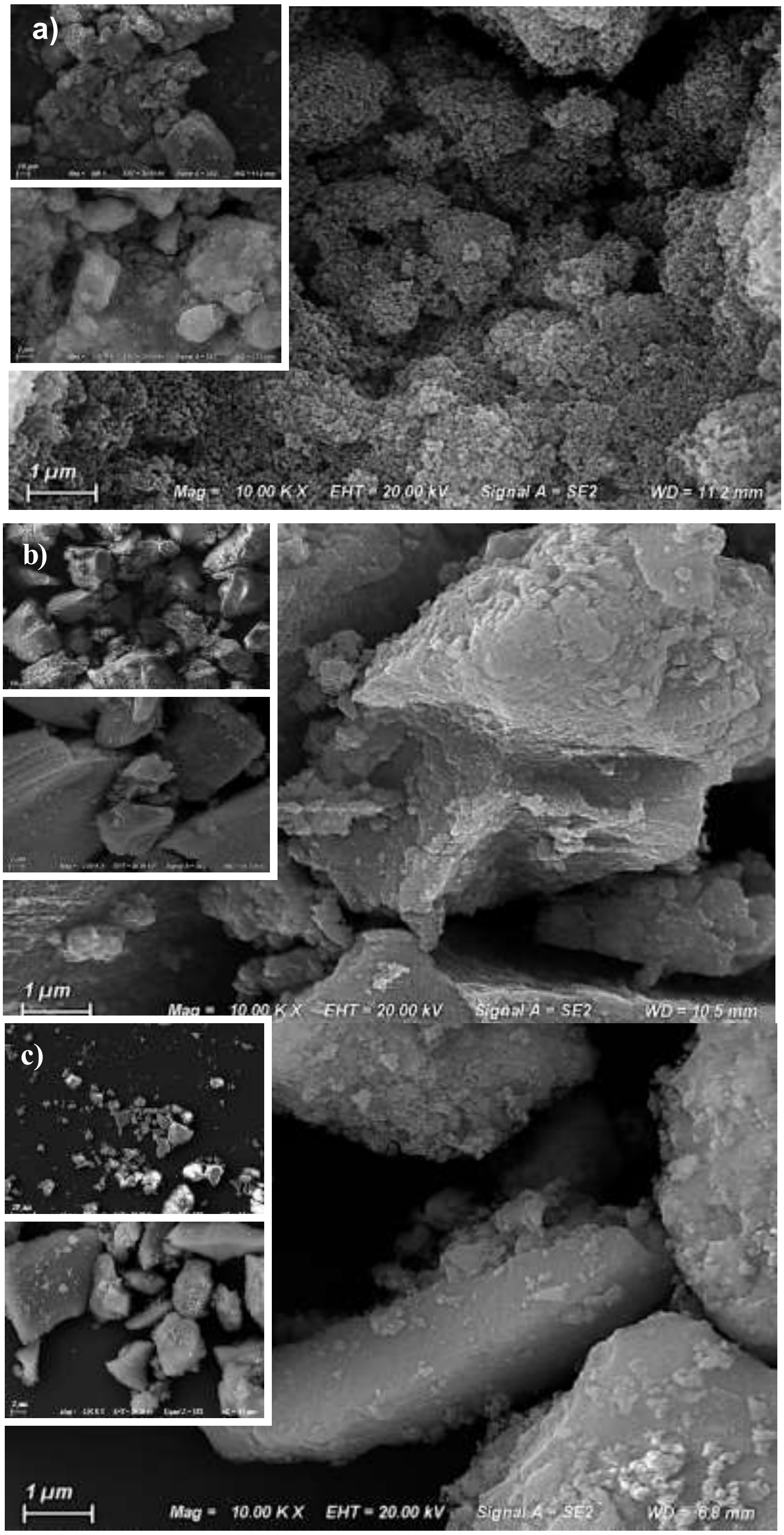

Figure S3. SEM images for a) Nb_2O_5-2; b) Nb_2O_5-4 e c) Nb_2O_5-6

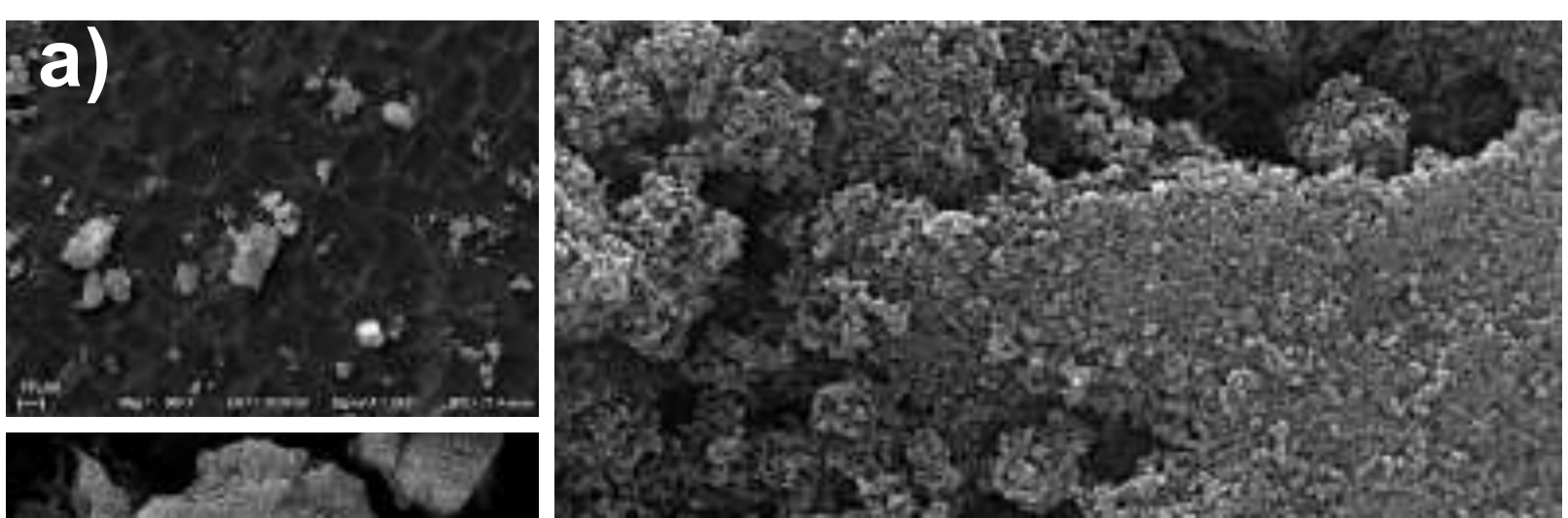

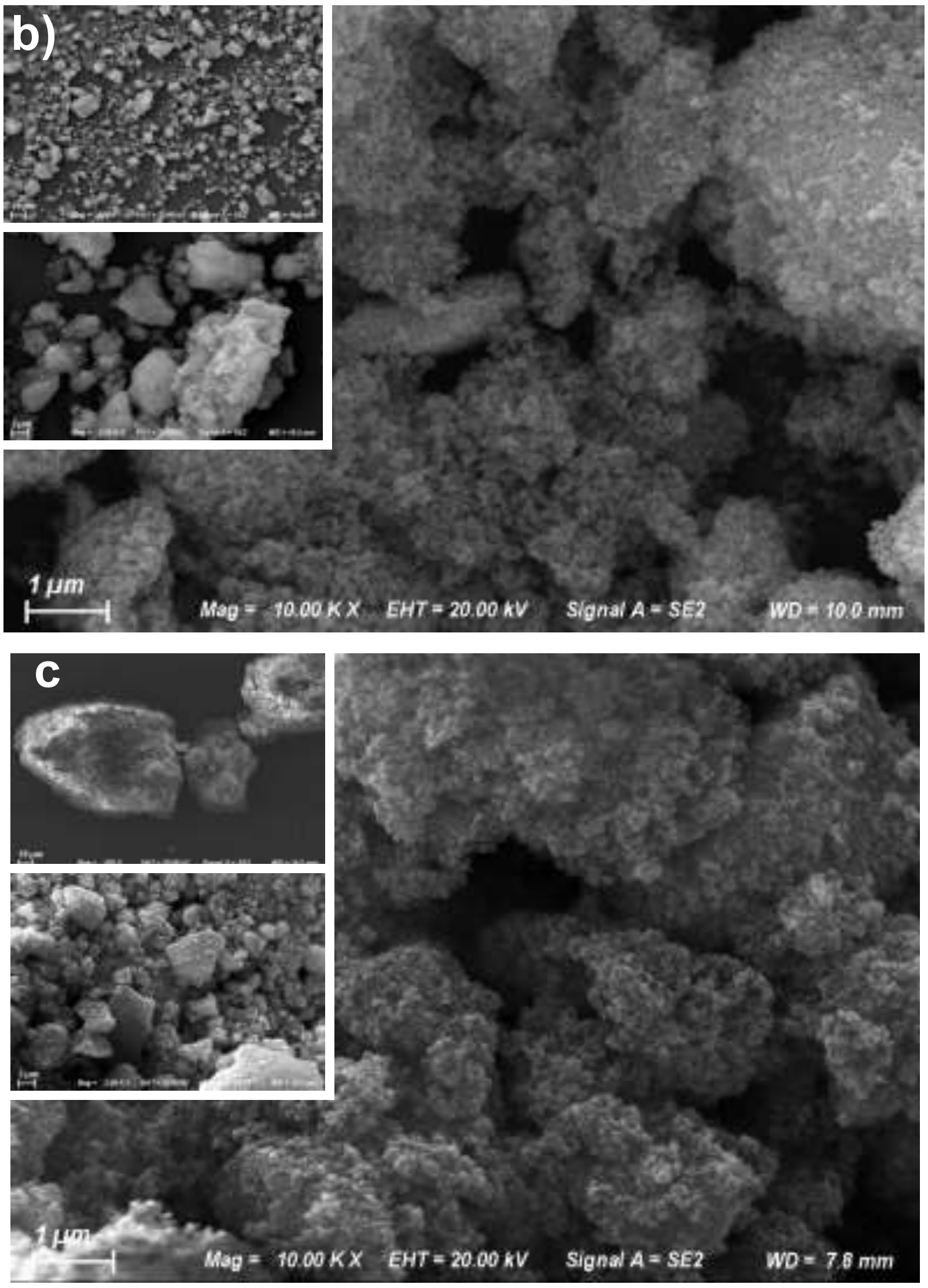

Figure S4. SEM images for a) Nb_2O_5-AP2; b) Nb_2O_5-AP4 e c) Nb_2O_5-AP6.

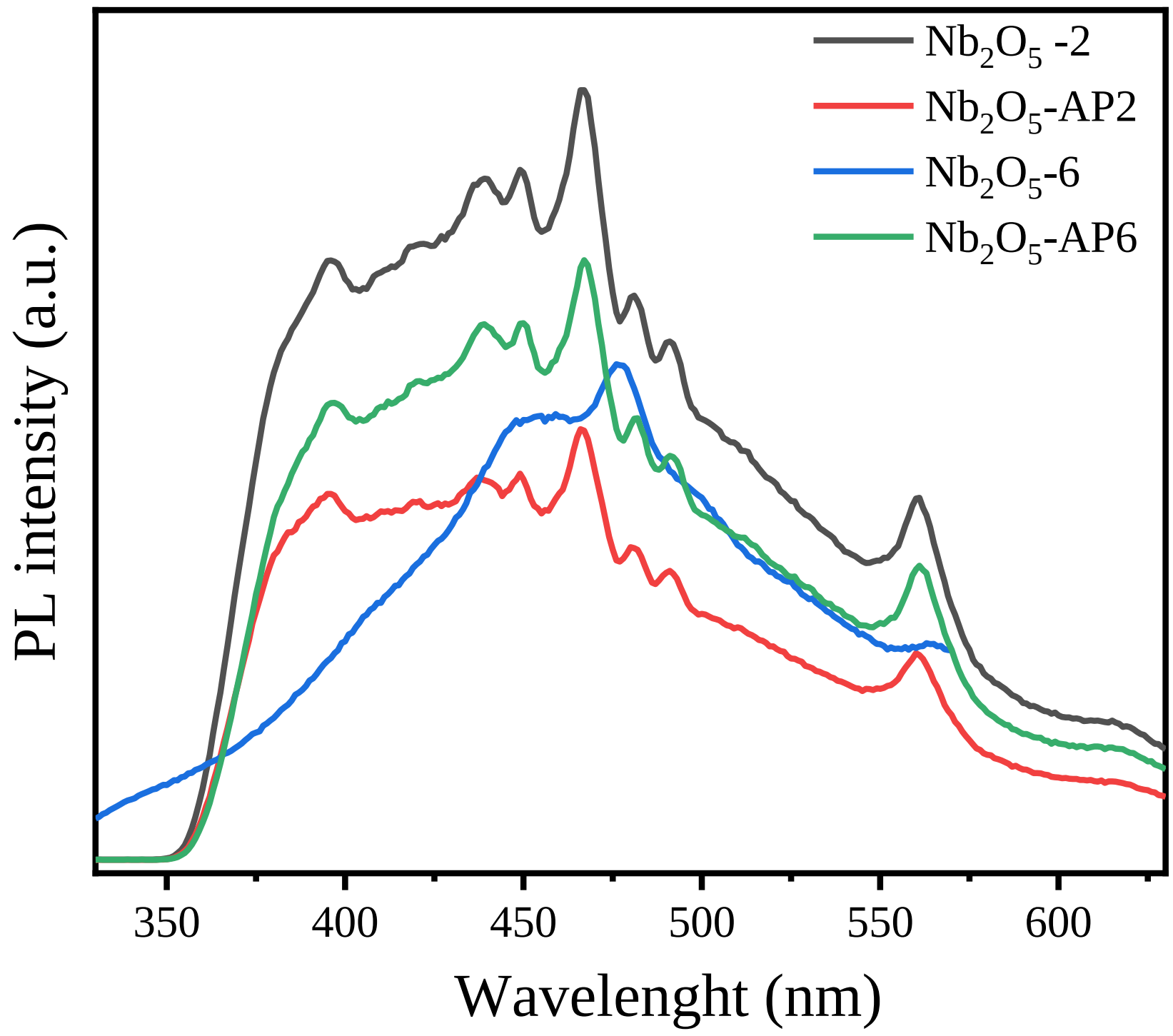

Figure S5. Photoluminescence emission spectra of Nb_2O_5 samples obtained using an excitation wavelength of 340 nm.
